# “Infantgram?” recruitment of infants to a clinical sleep study via social media

**DOI:** 10.1093/sleepadvances/zpae063

**Published:** 2024-10-03

**Authors:** Cathal O’Connor, Hannah O’Leary, Deirdre Murray, Geraldine B Boylan

**Affiliations:** Paediatrics and Child Health, Cork University Hospital, Cork, Ireland; Dermatology, South Infirmary Victoria University Hospital, Cork, Ireland; INFANT Research Centre, University College Cork, Cork, Ireland; INFANT Research Centre, University College Cork, Cork, Ireland; Paediatrics and Child Health, Cork University Hospital, Cork, Ireland; INFANT Research Centre, University College Cork, Cork, Ireland; Paediatrics and Child Health, Cork University Hospital, Cork, Ireland; INFANT Research Centre, University College Cork, Cork, Ireland

**Keywords:** sleep, electro-encephalography, infants, children, pediatric, recruitment, social media, atopic dermatitis, dermatology

## Abstract

**Study Objectives:**

This study aimed to outline the strategy and outcomes of a study team in recruiting participants for an infant sleep study via social media during the COVID-19 pandemic, to assess the feasibility of recruitment via social media, and to quantitatively and qualitatively explore parental satisfaction and perceptions of recruitment via social media.

**Methods:**

The assessing sleep in infants with early-onset atopic dermatitis by longitudinal evaluation (SPINDLE) study recruited infants with and without atopic dermatitis for a longitudinal study assessing sleep. Infants were recruited via social media and their parents were interviewed to explore their experience of recruitment via social media.

**Results:**

In total, 57 controls and 33 cases were recruited. Of the 45 controls recruited via social media, 43 (95.6%) were recruited via Instagram and 2 (4.4%) were recruited via Twitter. Of the seven cases recruited via social media, 6 (85.7%) were recruited via Facebook (via sharing of Instagram posts by third parties on Facebook) and 1 (14.3%) was recruited via Instagram. All (100%, *n* = 28) mothers recruited via social media who completed the full study were satisfied with this approach to recruitment. Specific reasons why mothers reported engaging following exposure to the social media posts included the benefit of additional health checks for their baby, the benefit to scientific advancement, and the opportunity for a stimulating outing following the COVID-19 lockdowns.

**Conclusions:**

Our experience highlights parents’ acceptance of recruitment via social media, the optimization of time and financial resources, and the benefit of using internet-based recruitment during a pandemic.

**Statement of Significance**

Recruitment of infants for sleep studies can be challenging, especially during periods of social distancing, such as during the COVID-19 pandemic. Specifically targeting mothers via social media can boost recruitment rapidly, at low or no cost, and can reach ethnically, socioeconomically, and educationally diverse groups. This study shows that recruiting infants for a sleep study via social media is a feasible, acceptable, and effective strategy for enhancing recruitment.

Recruitment of participants for scientific research studies can be challenging [1, 2]. There are multiple extra logistical and ethical considerations related to the recruitment of infants for research, particularly sleep studies [2]. Infant studies often require recruitment within a narrow timeframe, before they outgrow the inclusion criteria for age. Studies examining sleep in special populations, e.g. specific medical conditions, may have a short interval between disease diagnosis and study entry. It may be difficult to recruit healthy infants as controls if they are not required to attend healthcare settings regularly. Furthermore, the COVID-19 pandemic and social distancing policies have created unique obstacles to recruitment [3]. Given the ubiquity of social media use among women of childbearing age, the recruitment of infants for research studies by specifically targeting mothers via social media is an emerging practice [4], even outside the context of a pandemic. Advertisements via social media can be achieved rapidly, at low or no cost [5], and can reach ethnically, socioeconomically, and educationally diverse groups [6]. In addition, links to more detailed information such as parent/participant information leaflets can be provided easily. However, detailed protocols or strategies regarding the use of social media to recruit participants for infant studies have rarely been published, and even less so for infant sleep studies [7, 8]. This study aimed to outline the strategy and outcomes of a study team in recruiting participants for an infant sleep study via social media during the COVID-19 pandemic, to assess the feasibility of recruitment via social media, and to quantitatively and qualitatively explore parental satisfaction and perceptions of recruitment via social media.

## Methods

### Study background

The assessing sleep in infants with early-onset atopic dermatitis by longitudinal evaluation (SPINDLE) study (clinicaltrials.gov NCT05031754) recruited 6- to 8-month-old infants (*n* = 90) with moderate to severe atopic dermatitis (AD; *n* = 33) and age-matched control infants who did not have AD (*n* = 57) between December 2020 and May 2023. The aim of the SPINDLE study was to describe in detail the sleep architecture of infants with early-onset AD, compared to controls, by using EEG polysomnography, sleep actigraphy, and parental reporting [9]. The SPINDLE study involved four study visits to our center’s sleep and skin lab at 6–8 months, 9 months, 12 months, and 18–20 months. Control infants were initially recruited from ongoing studies in the research center. However, as the Covid pandemic took hold, there was dramatically reduced interaction with healthy control infants in person in healthcare settings. Therefore, a variety of creative strategies were utilized to optimize recruitment during the COVID-19 pandemic, as Ireland undertook strict mitigation measures to reduce the transmission of COVID-19.

### Recruitment strategies

**Figure 1.**
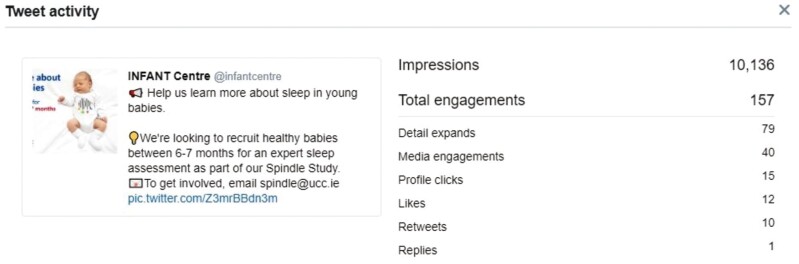
Post and analytics from Twitter, showing tweets and the number of impressions, engagements, etc.

**Figure 2.**
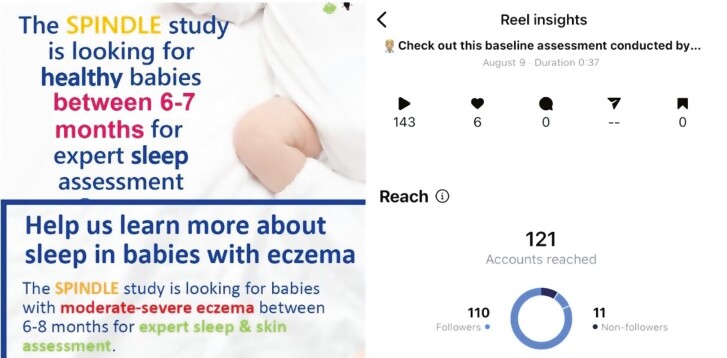
Poster and post analytics from Instagram. Top left—poster seeking controls. Bottom left—poster seeking cases. Right analytics for a video shared on Instagram.

Participants were sought for the full study (involving multiple sequential visits in person), or a questionnaire-only study for controls only (involving the completion of the brief infant sleep questionnaire monthly from 6 to 12 months). Participants who were unable to commit to the full in-person study were offered to complete questionnaires online to enhance data collection from control participants during the pandemic. Posters were circulated on our research center’s Instagram (~750 followers) and Twitter (~4500 followers) pages during recruitment drives in August and November 2021 (Figure 1–2). These social media accounts regularly post news about ongoing studies, publications, and awards from the research center. Our study researchers collaborated with our social media team to enhance the posts for maximal impact, by using language that was engaging and comprehensible for the target audience. Posts were left on the center’s pages indefinitely. Some posts included a video of a baseline assessment. Unpaid posts included the following information:

- An image of a baby with eczema (for the case group) or a sleeping baby with normal skin (for the control group).
- A headline to explain the purpose of the study e.g. “help us learn more about sleep in babies with eczema.”
- Text to provide further concise information on the study e.g. “the SPINDLE study is looking for babies with moderate-severe eczema between 6 and 8 months for expert sleep and skin assessment.”
- The university, research center, and study logos.
- Contact details by email.

There was significant engagement with followers and targeted local communities, with tweets receiving over 10 000 impressions, and videos being watched over 100 times.

### Recruitment feedback

A brief exit interview was held with mothers recruited via social media who completed the full study to assess their experience quantitatively and qualitatively. The interview was held in person at the final study visit. The following questions were asked verbally, and in a semi-structured manner, and responses were documented.

1. In terms of the social media posts you saw when you expressed interest in the study, were you happy with being recruited to the study in this way?
2. Were there any specific features about the social media posts or the study which led you to contact the study email?

Statements were written down verbatim and qualitative data were organized and coded on Microsoft Word. Themes were then identified, refined, and developed [10].

## Results

### Recruitment overview

**Figure 3.**
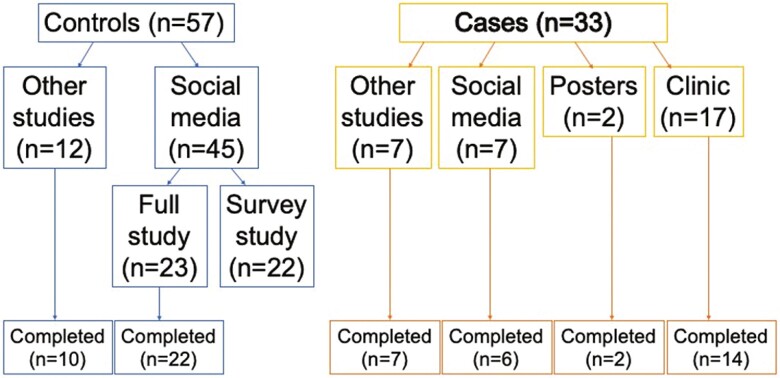
Flow chart showing methods of recruitment for cases and controls (*n* = 90), and retention to study completion.

In total, 57 controls (35 for the full study and 22 as part of a nested questionnaire-only study) and 33 cases were recruited (Figure 3). Of 57 controls, 45 were recruited via social media and 12 were recruited from other ongoing studies in the research center. Over the two recruitment drives in August and November 2021, 90 emails were received from parents of control infants seeking further information, with 45 parents agreeing to take part, giving a 50% (45/90) recruitment rate from those who expressed interest. Of the 45 controls (23 for the full study and 22 for the questionnaire-only component) recruited via social media, 43 (95.6%) were recruited via Instagram and two (4.4%) were recruited via Twitter. Of 33 cases, 17 were recruited via dermatology or pediatric outpatient clinics, seven were recruited from other ongoing studies in the research center, seven were recruited from social media, and two were recruited from physical poster advertisements in our pediatric outpatient department. Seven emails were received from parents of infants with AD seeking further information from social media, with all seven meeting eligibility criteria and agreeing to take part, giving a 100% (7/7) recruitment rate from those who expressed interest. Of the seven cases recruited via social media, 6 (85.7%) were recruited via Facebook (via sharing of Instagram posts by third parties on Facebook) and 1 (14.3%) was recruited via Instagram.

### Participant retention

Retention of participants during this longitudinal study was high. In total, 60/68 (88.2%) of participants enrolled in the full study completed the final visit at 18–20 months. Of controls, 22/23 (95.6%) of participants recruited via social media completed the final study visit, while 10/12 (83.3%) of other participants finished the study. Of the cases, 6/7 (85.7%) participants recruited via social media completed the final study visit, while 23/26 (88.5%) of other participants finished the study (Figure 3).

### Participant experience

A brief exit interview was held with mothers (22 controls, 6 cases) recruited via social media who completed the full study to assess their experience. All (100%, *n* = 28) mothers were satisfied with the approach to recruitment. Elaborating statements included *‘I felt that there was no pressure to participate as I was able to read the information about the study in my own time’* and *“I had been approached in the hospital about being involved in a different study and I had said no at that time as I was so exhausted after giving birth and I regretted saying no afterwards.” Mothers reported specific reasons why they engaged following exposure to the social media posts, such as the benefit of additional health checks for their baby (n = 23), the benefit to scientific advancement (n = 15), and simply the opportunity for a stimulating outing following the COVID-19 lockdowns (n = 12;*Table 1*). In terms of specific components of the posts which led parents to contact the study email, parents said that the images and*videos sparked interest in getting their child involved and made the study seem well-organized and professional.

**Table 1.** Themes From the Qualitative Statements From Parents Related to Motives for Expressing Interest From the Social Media Posts and Participating in the Study, With Illustrating Statements

Benefit of additional health checks for their baby
“I was interested in having extra assessments done for my baby as I was worried that his developmental checks might be missed during Covid.” “I was really impressed by all the testing that was being offered, totally for free.”
Benefit to scientific advancement
“Our older baby was in the neonatal unit so we know how important it is to contribute to research.” “We felt very lucky to have such a happy healthy baby, and this research project seemed like a very easy way to get her involved in science discovery from a super early age!”
Opportunity for a stimulating outing following the COVID-19 lockdowns
“Honestly, we were so delighted to have the possibility of venturing out of the house after lockdown, we were delighted to volunteer.” “The project looked enjoyable and well organized and we had missed out on other new baby social activities like mother-baby classes because of Covid.”.

Themes included the benefit of additional health checks for their baby, the benefit to scientific advancement, opportunity for a stimulating outing following the COVID-19 lockdowns.

Based on our experience from the study and from the feedback from parents, we have outlined our perceived benefits and disadvantages in Table 2.

**Table 2.** Benefits and Disadvantages of Using Social Media for Recruitment of Infants for Sleep Studies

*Advantages*
Broad and rapid exposure of potential participants to the advertisement of the study
Potential to engage participants who may not otherwise hear about or benefit from research
Certain demographics can be targeted to optimize exposure
Less effort and more time/resource efficient to recruit
Pandemic-proof reduced the need to perform in-person eligibility assessments
Cheap/free
More time/information for parents to reflect and decide on participation in the study
More likely to retain participants in the study if truly interested
*Disadvantages*
May miss participants who do not use social media, eg, disadvantaged SEC groups
Parents may misunderstand the nature of the research study
Potential for contact from individuals who are not interested in the study
Potential for scam participants
May attract participants with sleep disorders seeking assessment/treatment

SEC, socioeconomic.

## Discussion

This study highlights the strategy, feasibility, acceptability, and success of recruiting infants for a sleep study via social media. Previous research has provided a structured approach to recruitment via social media that can be adopted according to the study design [11]. While successful recruitment via social media has been described in other studies, traditional methods of recruitment are reported to yield more participants, although social media may be more effective for observational studies [12]. Therefore it is unlikely that recruitment via social media will entirely replace recruitment via conventional pathways and is likely best used as a complementary method to enhance efficiency. There is little specific regulatory guidance and limited bioethics literature to guide researchers regarding the ethical issues that recruitment via social media raises, although frameworks have been suggested to direct researchers [2, 12, 13]. Key ethical issues related to sampling bias, equality of access, recruitment effectiveness, target group vulnerability, privacy and data security, transparency, and information and consent [13]. Sampling bias can occur as potential participants may already follow the research center’s accounts and therefore be interested or willing to participate in studies related to the field of interest. Research centers which have minimal presence on social media may struggle to recruit using unpaid ads.

Our experience of recruitment via social media in the context of the COVID-19 pandemic was positive. The most obvious benefit was rapid and broad exposure to potential participants within our area, especially for control infants. If the participation of particular socioeconomic groups is desired, then posts or advertisements can be targeted using algorithm-based platforms [3], although this may increase costs related to paid advertising, rather than the unpaid posts which we used. In our study, expressions of interest were responded to by email or phone, and study visits were performed in person, which avoided problems related to potential scam participants. Parents of infants with sleep problems may be motivated to seek recruitment as a control, due to hopes of having a sleep assessment or treatment, which may skew the control data. Social media may also have a role in augmenting retention [14], as participants recruited via social media have actively expressed interest and willingness to engage in a study, compared to other opportunistic means of recruitment such as being approached on postnatal wards. As half of the control participants recruited via social media were involved only in the online questionnaire-only component of the study, recruitment via social media may be helpful for studies seeking to collect data from participants for virtual or online-based assessments.

In our study, we noted that Instagram was the social medium most likely to generate interest for control participants, while Facebook was most likely to generate interest for cases (with posts shared from Instagram by third parties). Facebook seems to be a common platform for people with common interests to share recruitment posts about a study. Unpaid posts on Facebook, or in specific Facebook groups with permission, may represent a helpful strategy to identify niche participants. Instagram posts and reels are shared broadly on the home screen. Despite reaching many accounts on Twitter with posts, only two participants were recruited from Twitter, despite the fact that the research center had many more followers on Twitter (4500) than on Instagram (750). This may relate to Twitter being considered a more professional or academic social medium than Instagram, with less public exposure. The use of images embedded in social media posts is known to significantly enhance engagement [15], and the use of videos using the Instagram “reel” function may further enhance interest, especially if they include a demonstration of what the study involves. Limitations of this study include the focus on Instagram and Twitter and the lack of other platforms such as TikTok. Centers that do not have a communications team might require additional input to create impactful posts.

As a relatively novel approach to recruitment, researchers may be skeptical of the robustness of using social media for participant recruitment in healthcare research. Our experience in SPINDLE highlights parents’ acceptance of recruitment via social media, the optimization of time and financial resources, and the benefit of using internet-based recruitment during a pandemic. Future sleep studies in infants and children should consider using social media as part of the recruitment strategy.

## Supplementary Material

zpae063_suppl_Supplementary_Figures_S1

## Data Availability

The dataset generated from this study will be available upon reasonable request from the corresponding author (COC). The data will not be publicly available due to consideration for information that could compromise research participant privacy or consent.
